# The Indispensability of Snail Control for Accelerating Schistosomiasis Elimination: Evidence from Zanzibar

**DOI:** 10.3390/tropicalmed7110347

**Published:** 2022-11-03

**Authors:** Mtumweni Ali Muhsin, Xinyao Wang, Fatma Mohammed Kabole, January Zilabumba, Kun Yang

**Affiliations:** 1 School of Medicine, Jiangnan University, Wuxi 214122, China; 2 Neglected Tropical Disease Control Programme, Ministry of Health, Mnazi Mmoja, Zanzibar 16108, Tanzania; 3 Jiangsu Institute of Parasitic Diseases, Wuxi 214064, China; 4 Key Laboratory of National Health and Family Planning, Commission on Parasitic Disease Control and Prevention, Jiangsu Provincial Key Laboratory on Parasite and Vector Control Technology, Jiangsu Institute of Parasitic Diseases, Wuxi 214064, China; 5 School of Public Health, Nanjing Medical University, Nanjing 211166, China

**Keywords:** *Schistosoma haematobium*, mass drug administration (MDA), snail control, Zanzibar, joinpoint regression model (JRM)

## Abstract

Schistosomiasis is a serious and neglected global tropical disease, affecting upwards of 230 million people, with more than 95% of infections concentrated in Africa. For many years, the main schistosomiasis control strategy in Africa focused on mass drug administration (MDA). The aim of this study was to compare the difference between MDA alone and alongside another intervention, namely snail control, by exploring effective measures for eliminating schistosomiasis. Retrospective data of human prevalence on *Schistosoma haematobium* and major control measures were collected from the China-Zanzibar-WHO Cooperation Project for Schistosomiasis Elimination (CZW) and the Zanzibar Elimination of Schistosomiasis Transmission (ZEST) project since 2012. The optimal order polynomial regression fitting model and joinpoint regression model (JRM) were used to analyze trends in schistosomiasis prevalence and the consistency of change points with strengthening of the control measures. In Unguja Island, the main control measure was MDA, and prevalence decreased to a nadir in 2019, and then rebounded. The R^2^ value of the optimal fitting model was 0.6641. There was a single JRM changepoint in 2019, the annual percent change (APC) was −19.3% (*p* < 0.05) from 2012 to 2019, and the APC was 59.7% (*p* > 0.05) from 2019 to 2021. In Pemba Island, the main control measures until 2016 was MDA, while integrated measures of MDA and snail control were implemented from 2017, the prevalence continuously decreased, and the R^2^ value was 0.8673. There was also a single JRM changepoint in 2017, the APC was −22.2% (*p* < 0.05) from 2012 to 2017, and was maintained at −8.6% (*p* > 0.05) from 2017 to 2021. Our data indicate that, while it is challenging to eliminate schistosomiasis by MDA alone, integrated measures, including both MDA and snail control, can prevent reinfection and help to eliminate the diseases in Africa.

## 1. Introduction

Schistosomiasis is a group of diseases caused by parasitic worms of the genus *Schistosoma*. These blood-dwelling flukes have a complex life cycle involving freshwater snail intermediate hosts, and the transmission of the parasite is governed by social-ecological systems and is intimately linked with conditions of poverty [1,2]. Schistosomiasis endangers human health and affects social and economic development in 78 countries and regions in Africa, Asia, South America, and the Middle East. More than 230 million people are infected with schistosomes worldwide, with more than 95% of infections concentrated in Africa [3,4,5]. Human schistosomiasis is caused by infection with one of five species of schistosomes, namely *Schistosoma mansoni*, *Schistosoma japonicum*, *Schistosoma haematobium*, *Schistosoma intercalatum*, or *Schistosoma mekongi*, with the first three species having the greatest clinical and socio-economic importance [6]. Four of the five species, except for *S. haematobium*, cause hepatosplenic and intestinal schistosomiasis, in which the parasitic worms inhabit the mesenteric veins, whereas *S. haematobium* resides in the venous plexuses of the urinary bladder and causes urogenital schistosomiasis [7,8], the numerous complications of which include bladder cancer [9,10]. Schistosomiasis is distributed in most countries in Africa, including Kenya, Ethiopia, Egypt, and Zanzibar [11].

It has been almost 100 years since schistosomiasis was first chronicled as a public health concern in Zanzibar [12]. Historically, the islands represented an area with a high prevalence of severe disease caused by *S. haematobium* [13,14]; however, there was a lack of control interventions before 1986. *Bulinus* spp. is a hermaphroditic group of freshwater snails of about 30 species that all can transmit *S. haematobium* [5]. Control interventions were first initiated in Pemba Island [15], followed by intermittent appraisal of their outcomes, which demonstrated the efficacy of the treatment of schistosomiasis with the praziquantel [16]. The “Piga Vita Kichocho” (“War against Schistosomiasis”) project, which started in 2001 in Unguja and Pemba, and involved treating school-aged children annually, followed by sentinel surveillance from 2004 to 2006. Praziquantel is a highly effective drug that remains the treatment of choice for all forms of schistosomiasis infection [17], and is also active against a broad range of parasitic helminths, including clonorchiasis, opisthorchiasis, tapeworm infections, cysticercosis, and hydatid disease [18].

Snail control, mainly by molluscicide, is the cornerstone of schistosomiasis control before the strategy for morbidity control, and has contributed to many successful control outcomes [19,20]. Niclosamide is the only recommended molluscicide for snail control by the World Health Organization (WHO) [21]. It was mentioned at the Sixth International Congress on Tropical Medicine and Malaria held in Lisbon in 1958, and was found to have a high molluscicidal efficacy and low toxicity to animals, and there was no evidence of poisoning caused by faulty operation or by accidental ingestion [22]. From the 1990s, a large quantity of niclosamide was used in China, which made a great contribution to the control of schistosomiasis. The most used molluscicides were 50% wettable powder of niclosamide (WPN), 70% WPN, and 26% suspension concentrate of metaldehyde and niclosamide (MSCN) in China. Furthermore, over the past few decades, there have been few reports about the accumulation of niclosamide in the environment or about any negative ecological impact [23,24].

The Zanzibar Elimination of Schistosomiasis Transmission (ZEST) project began in 2012 and involved biannual community mass drug administration (MDA) with praziquantel in Unguja and Pemba. ZEST was an intervention research project funded by the Schistosomiasis Consortium for Operational Research and Evaluation (SCORE) to assess whether a small amount of snail control and behavior change, together with MDA, would have a larger impact on schistosomiasis. The project aimed to eliminate schistosomiasis in both Unguja and Pemba [25,26].

In 2014, the World Health Organization (WHO) signed a tripartite memorandum of understanding (MOU) with China and Zanzibar, paving the way for implementation of a pilot schistosomiasis elimination program in Zanzibar, the China-Zanzibar-WHO Cooperation Project for Schistosomiasis Elimination (CZW). The objectives of the CZW program were to control and eliminate schistosomiasis, based on the experience of the schistosomiasis control from China, specifically including snail control using molluscicides [27]. Unlike ZEST, the intervention methods included MDA and snail control interventions in Pemba from 2017–2021 [28]. The CZW program used 26% MSCN, which was provided by Nanjing AI Jin Chemical Co., Ltd. (Nanjing, China), to control the snails, and the dose of molluscicide was 2 g/m^3^. The ponds and streams with positive snails used MSCN once a year for controlling the snails.

The time trend analysis is an important component of epidemiological research [29]. The traditional regression models generally fit and evaluate the overall trends in disease distribution over the entire study time period, and cannot show changes occurring at specific time points. In 1998, Kim first proposed the joinpoint regression model (JRM) [30], the core idea of which was to establish segmented regression, according to the time characteristics of disease distribution. They divided the research time into different intervals through several connection points, and performed trend fitting and optimization for each interval, so as to provide a detailed evaluation of time interval-specific disease variable characteristics across the investigated time scale. JRM is a trend analysis method that uses statistical methods to explore turning points. It divides the long-term trend into several statistically significant trend segments through model fitting, where each segment is described by a continuous linear line. JRM incorporates a traditional regression model, but performs analytical functions that cannot be achieved by traditional methods [31]. In this study, historical data on human *S. haematobium* infection prevalence, as well as main control measures, were collected from the CZW and ZEST projects from 2012 to 2021. The aim of this study was to compare the effects of different interventions using MDA with or without snail control, to explore the effectiveness of measures for the elimination of schistosomiasis.

## 2. Materials and Methods

### 2.1. Study Area

Zanzibar is part of the United Republic of Tanzania and consists of two main islands, Unguja and Pemba, as well as several islets. There are two wet seasons each year: the long rains from March to June, while the short rains are between October and November. The average annual temperature ranges between 23 °C and 32 °C. Islam is the predominant religion. The main economic activities include seawater fishing and cash crop production. Unguja has seven districts (North A, North B, Central, West A, West B, South, and Urban) and more than 300 shehias. Pemba comprises four districts (Micheweni, Wete, Chake, and Mkoani), subdivided into more than 100 shehias. *S. haematobium* is endemic in Zanzibar, and its intermediate snail host is *Bulinus globosus* [32,33]. In Unguja, schistosomiasis is endemic in North A, North B, Central, West, and a part of the Urban districts, whereas it is endemic in all four districts of Pemba [34].

### 2.2. Study Design

This was a retrospective quantitative study that reviewed secondary data generated from randomly selected shehia, as well as urine samples from schoolchildren. The study focused on data from schoolchildren in Pemba and Unguja, as they are in the age range where schistosomiasis is concentrated. A clustered sampling method was used to select the monitoring sites. During data collection, a random sampling procedure was used to identify schoolchildren for the collection of urine samples. Urine samples were transported to the laboratory, and a 10 mL syringe was then used to extract 10 mL of urine, which was then filtered. The cu was repeatedly shaken to include eggs that have a tendency to collect at the bottom. The filter device was prepared with the filter taken out and was placed on a glass slide before microscopy.

As the samples from both islands were collected after an MDA program, they served as reliable indicators of progress in the containment of schistosomiasis; however, the two islands used different intervention methods to target the disease; a snail control program was implemented under the CZW program in Pemba, alongside MDA, while in Unguja, only MDA was implemented. Thus, there were two branches of intervention, which were further branched in Pemba, due to the initiation of the snail control through the CZW project in 2017.

**Figure 1:**
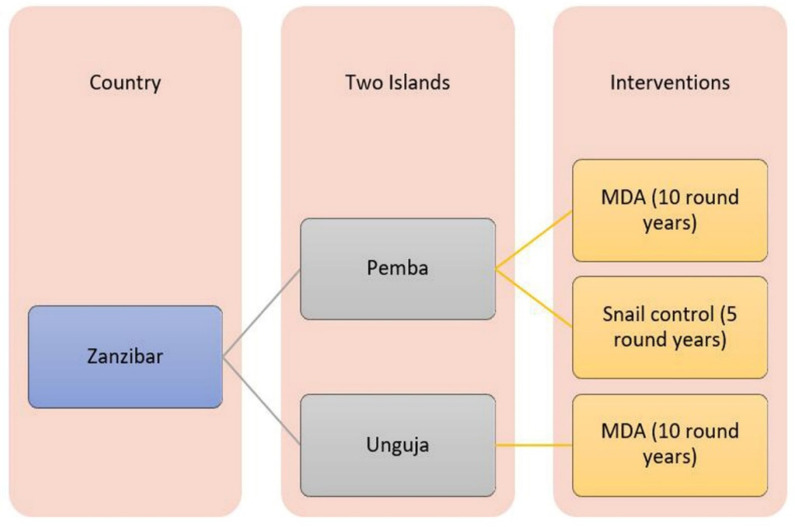
Data collection framework.

A nonlinear relationship between the number of rounds of MDA and schistosomiasis prevalence was assumed. The number of rounds of MDA was the independent variable and infection prevalence was the dependent variable. As surveillance was conducted once per year, MDA was also completed once each year. Therefore, the year was substituted for number of rounds, and MDA was implemented equally on both islands (Figure 1).

### 2.3. Data Collection

Secondary data from the ZEST project were reviewed. These data were generated over 10 years by two sections, comprising monitoring sites in the community and in schools. Data from schoolchildren (age range of 9–15 years) from 45 schools were collected in Unguja and Pemba. Urine samples were collected from 100 children in each school, totaling 4500 people per island per year, and the mean prevalence per island per specific year was determined based on infection data from the schoolchildren. The CZW represented a different intervention method, as snail control was used as part of this program to target the disease in Pemba since 2017, allowing for a comparison of the two islands according to whether or not snail control was implemented.

### 2.4. Data Analysis

Only one independent variable was investigated in this study; therefore, multicollinearity and a potential drawback were not investigated when using regression analysis. As the trend of schistosomiasis prevalence is non-linear [35], significant relationships between the two variables (number of MDA and prevalence, as independent and dependent variables, respectively) were analyzed and model formulae were generated using SPSS 20.0 (International Business Machines Corporation). The SPSS software outputs were tables of coefficient values for the regression equations (models) and the results of the tests of significance for the independent variable, including R^2^ and *p* values [36]. Optimal order polynomial regression fitting models were obtained by comparing the R^2^ values of the regression models. Then, Excel 2016 statistical software was used to generate graphs for Unguja and Pemba, described by the equations governing the change in prevalence according to year. Curve formulae were derived and the slopes were used to compare the rates of change in disease prevalence in Pemba and Unguja.

JRM can be divided into linear and log-linear models, and dependent variables can include the number of events (number of cases), morbidity/mortality ratio, and composition ratio, among others. Data distribution types include normal distribution, exponential distribution, or Poisson distribution [37]. If the dependent variable obeys the normal distribution, a linear model is selected, whereas if the dependent variable follows an exponential or Poisson distribution, the log-linear model is selected [31]. Models were analyzed for statistically significant turning points using the Monte Carlo permutation test, the corresponding *p* and t values were calculated, and the best model was selected according to the Bayesian information criterion [38]. Trend change indicators are expressed as annual percent change (APC) and average annual percent change (AAPC), where APC > 0 indicates that the rate increased year by year, APC < 0 indicates that the rate decreased yearly, AAPC > 0 shows that the rate increased on average every year, and AAPC < 0 that the rate decreased every year on average [39]. JRM was established using the schistosomiasis infection rate of the two islands as the dependent variable (y) and the year as the independent variable (x). Joinpoint Regression Program 4.9.1.0 (Statistical Research and Applications Branch, National Cancer Institute) software was used to analyze the trends in schistosomiasis prevalence on the two islands after different measures were adopted from 2012 to 2021.

## 3. Results

### 3.1. Basic Information

Prevalence data for the different districts of Unguja and Pemba Island were successively collected from 2012–2021 (Table 1). The mean prevalence rates for districts in Unguja Island were 3.5%, 5.7%, 4.4%, 6.9%, 3.4%, 1.6%, 2.4%, 0.7%, 3.3%, and 3.0% from 2012–2021, respectively, while those of the districts in Pemba Island were 8.4%, 8.5%, 5.5%, 4.4%, 5.5%, 1.7%, 3.4%, 1.7%, 2.7%, and 1.7%. These data were generated over 10 years from the school monitoring sites. Data from children aged 9–15 years were collected from 45 schools each in Unguja and Pemba; urine samples were collected from 100 children per school, totaling 4500 people per island per year. As each school generated data, we focused on the mean prevalence values per island in each specific year.

### 3.2. Regression Analysis of the Relationship between MDA and Prevalence

**Figure 2:**
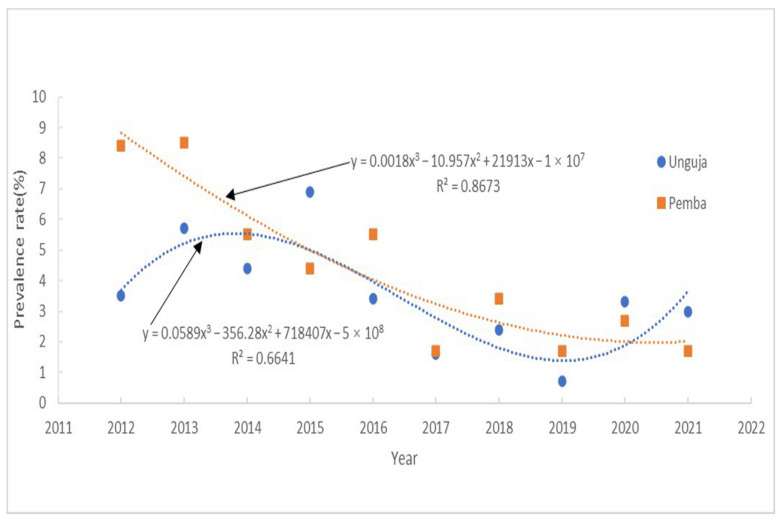
Testing curves showing schistosomiasis prevalence in Unguja and Pemba according to year.

SPSS software was used to generate scatter plots illustrating the relationships between the independent variable (year) and dependent variable (prevalence of schistosomiasis). Ordinary least squares regression analysis indicated a non-linear relationship between the two variables [40,41]. Polynomials of different orders were generated using SPSS software (Table 2). A comparison of the R^2^ values indicated that a third order polynomial distribution was most consistent with the changes in the data curve. R^2^ values represent model fit and are important indicators of the goodness of fit of a linear equation, reflecting the ability of the regression model to explain the variation of the dependent variable. In this model, R^2^ was 0.6641, indicating the goodness of fit of the model, where 66.41% of the variation can be explained by a linear relationship with MDA in Unguja. Year (round of MDA) was correlated with prevalence of schistosomiasis in Unguja (Figure 2). From the test curves, it can be observed that during MDA application in Unguja from 2012–2021, disease prevalence first increased, reached a peak in 2014, subsequently reached a nadir in 2019, and then rebounded. Hence, the testing curves indicated cyclical variation in schistosomiasis prevalence.

Furthermore, year (round of MDA) was also correlated with schistosomiasis prevalence in Pemba (Figure 2), with an R^2^ of 0.8673, indicating that 86.73% of the variation in the prevalence could be explained by the relationship with MDA in Pemba. The generated curved showed that the prevalence dropped sharply after the introduction of MDA in Pemba from 2012–2016. Furthermore, after the implementation of both snail control and MDA, the prevalence began to plateau and then decline slowly from 2017–2021.

### 3.3. JRM Analysis of Trends in Prevalence

**Figure 3:**
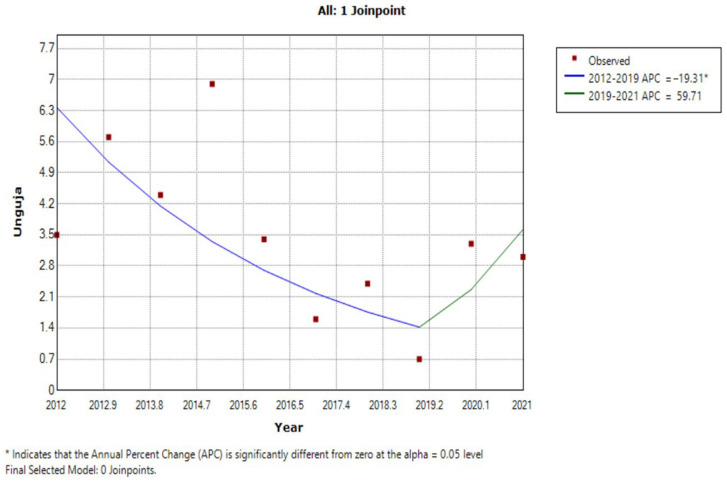
JRM for the trend in schistosomiasis prevalence in Unguja from 2012 to 2021.

The results of the JRM analysis of data from Unguja showed that schistosomiasis prevalence decreased at a mean rate of −19.3% per year from 2012 to 2019 (Z = −2.6, *p* = 0.048), and increased at 59.7% per year from 2019 to 2021 (Z = 0.8, *p* = 0.482). Thus, the schistosomiasis infection rate showed an inflection point in 2019, dividing the data into two time periods, 2012–2019 and 2019–2021, where the JRM curve first decreased and then increased. The rate of schistosomiasis prevalence decrease from 2012 to 2019 was significant (APC = −19.3%, *p* < 0.05), while the increase in schistosomiasis prevalence rate from 2019 to 2021 was not significant (APC = 59.7%, *p* > 0.05) (Figure 3).

**Figure 4:**
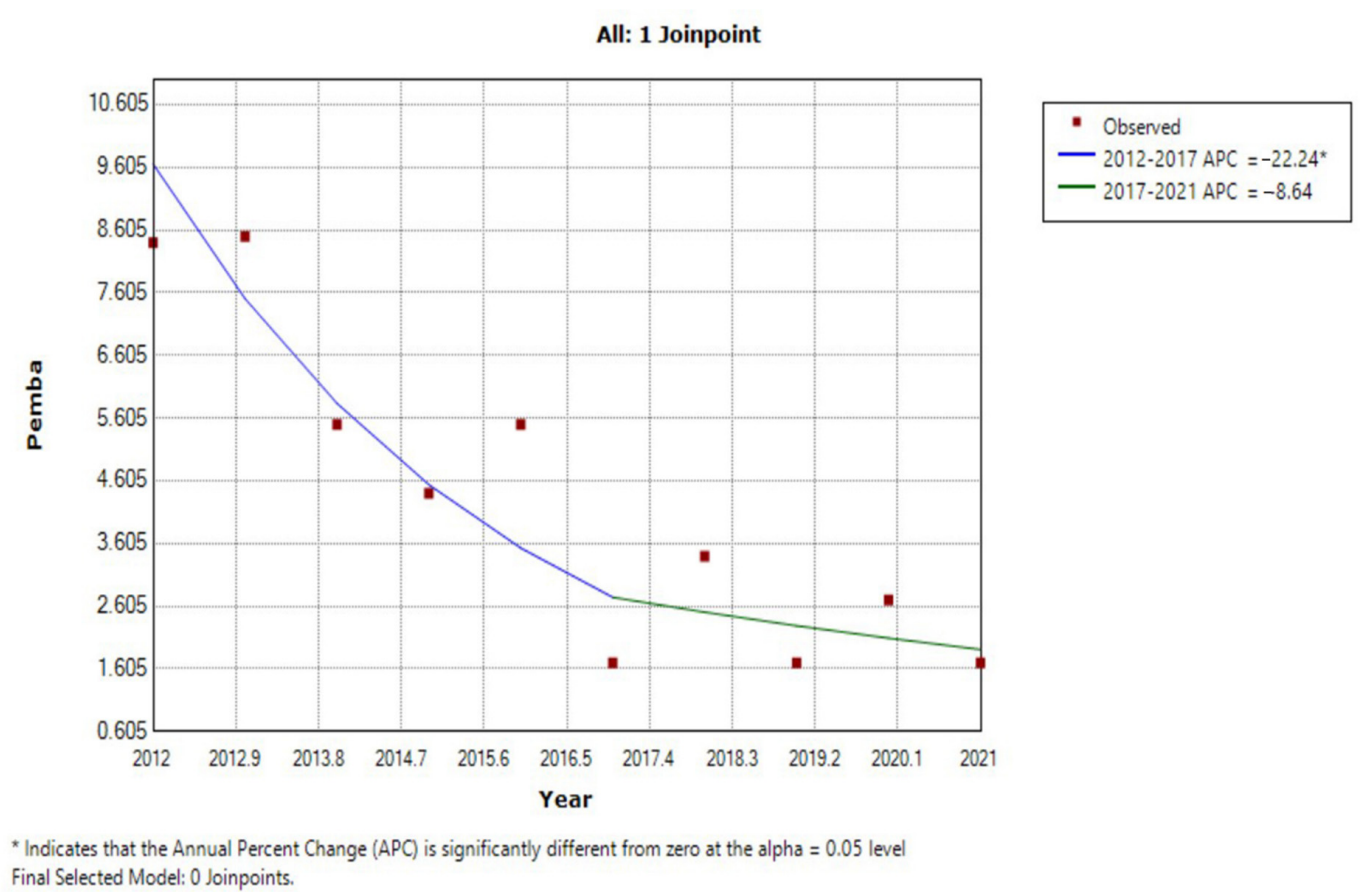
JRM for the trend in schistosomiasis prevalence in Pemba from 2012 to 2021.

In Pemba, schistosomiasis prevalence decreased at a mean rate of −22.2% per year from 2012 to 2017 (Z = −3.0, *p* = 0.029, *p* < 0.05), and then decreased at −8.6% per year from 2017 to 2021 (Z = −0.8, *p* = 0.474, *p* > 0.05). The schistosomiasis infection rate showed an inflection point in 2017, dividing the data into two time periods, 2012–2017 and 2017–2021. The JRM curve first decreased rapidly, followed by a more gradual decline. The rate of schistosomiasis prevalence decrease from 2012 to 2017 was significant (APC = −22.2%, *p* < 0.05), while that from 2017 to 2021 was not (APC = −8.6%, *p* > 0.05) (Figure 4).

## 4. Discussion

This study was based on data from the ZEST and CZW projects on schistosomiasis prevalence after different interventions, which we subjected to statistical analyses. Schistosomiasis is a water-borne disease and individuals can be infected through contact with water containing cercariae [42,43]. More cercaria shed from *Bulinus* intermediate host species of *S. haematobium* will be present in areas with higher numbers of water bodies. Hence, the prevalence of schistosomiasis is higher in districts where people have more contact with water and where ponds are closer to schools. Schoolchildren of 9–15 years old are more likely to be affected by schistosomiasis than people in any other age range [25,26,44]; therefore, we analyzed the prevalence rates in schoolchildren aged 9–15 years from 45 schools in Unguja and 45 in Pemba, including urine samples collected from 100 children in each school, corresponding to 4500 people per island per year. Although schoolchildren as the surveillance population can more intuitively reflect the effects of the control measures, the approach has certain limitations for the analysis of the entire region without considering all different age groups.

Snail control is recognized by the WHO. To guide member states to conduct effective schistosomiasis elimination, in February 2022, the WHO issued the “WHO guideline on control and elimination of human schistosomiasis”. The guideline development group agreed that a reduction in *Schistosoma* transmission via snail control is technically feasible in some settings. The evidence of the effectiveness of focal snail control supports the use of this intervention as a complementary measure to reduce the prevalence of schistosomiasis [45].

The praziquantel of MDA achieved 100 % geographical coverage and above 75% epidemiological coverage in Zanzibar. As surveillance was conducted in Zanzibar after MDA each year, our data included a mixture of regions where praziquantel was administered either once or twice annually; therefore, we used year, rather than number of MDA rounds, for our analyses, disregarding the number of rounds of medication administration. Importantly, all MDA programs were implemented equally on both islands; hence, analysis of the number of rounds as years rather than number of treatments is not expected to impact the statistical analysis. We hypothesized that there would be a non-linear relationship between the year of MDA and schistosomiasis prevalence, and so regression analysis was used to test the hypothesis. The results showed that there was a non-linear relationship between the two variables.

We collected successive prevalence rate data from different districts in Unguja and Pemba Island from 2012–2021. However, the sample size of the study was large populations from these two islands, and the data have been continuously researched for ten years, indicating that they were accurate and reliable. We assumed that all parameters not included in our analysis were the same over time and among districts. For instance, the timing of snail control interventions and surveys were not coordinated. There were also differences in the intervention approach between private and public schools, where the data from Pemba included both private and public schools, and those from Unguja included only public schools. The two projects (ZEST and CZW) analyzed in this study implemented not only snail control and MDA, but also other measures, such as behavioral interventions; however, the relevant literature reports demonstrated that the availability of a latrine and washing hands before eating did not significantly lower the risk of helminth infections in the study population [26]. There were also factors beyond the control of individuals that often impeded behavioral change, such as local and regional socioeconomic and political circumstances; for example, schistosomiasis may be given low priority, community participation and regional or national support may be inadequate, and enabling and reinforcing factors may be lacking [46]. In this study, we only performed statistical analysis on three factors: snail control, MDA, and location. In the future, we will study other factors that influence the seasonality of schistosomiasis infection, including behavioral aspects, water, sanitation, and hygiene (WASH), as well as numerous other factors that could potentially influence the subsequent schistosomiasis infection rates.

Although praziquantel can be used to treat patients with schistosomiasis, there are persistent hotspot areas where disease is intractable, notably in Unguja. Patients can be reinfected with schistosomiasis after recovery following MDA [47]. Praziquantel clears very quickly via in vivo metabolic processes in humans [48], while cercaria can survive for 72 h to enter the human host. The six-month spacing of the preventive chemotherapy (PC), and most of the time beyond planned six month rounds of PC provided ample opportunity for re-infection in Zanzibar. Furthermore, during every round of PC, some individuals were reluctant to take praziquantel, and these people remain sources of new miracidia for snails present along river shores and in ponds. Once snails are infected, the sporocyst develop into cercaria in order to wait for a human host to enter and restart the cycle. Our results show a clear cyclical variation between MDA and schistosomiasis prevalence, indicating that the prevalence declines slowly. Some reports have shown that, despite the rise in PC campaigns, as many people suffered from schistosomiasis today as they did 50 years ago. Snail control can complement PC by reducing the risk of transmission from snails to humans [49,50,51].

The two islands included in this study implemented different intervention methods to target schistosomiasis. Pemba had a strong snail control program under the CZW project, alongside MDA, while Unguja only implemented MDA. Thus, we were able to analyze the results of a dichotomy of two branched interventions, which were further branched in Pemba, due to snail control timing, as this approach was first recorded in 2017. The results of JRM showed that, in Unguja, schistosomiasis prevalence decreased at a mean rate of −19.3% per year from 2012 to 2019 (Z = −2.6, *p* = 0.048, *p* < 0.05), and increased at 59.7% per year from 2019 to 2021 (Z = 0.8, *p* = 0.482, *p* > 0.05). In Pemba, schistosomiasis prevalence decreased at a mean rate of −22.2% per year from 2012 to 2017 (Z = −3.0, *p* = 0.029, *p* < 0.05), and decreased at −8.6% per year from 2017 to 2021 (Z = −0.8, *p* = 0.474, *p* > 0.05). Therefore, the average annual prevalence of schistosomiasis in the population initially decreased by approximately 20% in both populations under the MDA intervention. A bottleneck period is encountered in later stage MDA, and schistosomiasis prevalence will periodically rebound and increase. Our findings indicate that snail control has a significant impact when combined with MDA. The application of a single MDA program can reduce schistosomiasis prevalence, while integrated measures including both MDA and snail control can prevent reinfection and eliminate these diseases in Africa.

This study has some limitations. The data analyzed were secondary findings from the ZEST and CZW projects, and the design was retrospective, which makes it inherently subject to certain biases [52]. Furthermore, due to the relatively small dataset analyzed in this study, some of our results were not statistically significant; therefore, we will continue to monitor the population to observe long-term effects.

From previous related studies, it is clear that the application of MDA alone for the prevention and control of schistosomiasis in Africa cannot completely eliminate the disease; hence, we should not only treat patients, but also pay attention to comprehensive management methods, such as snail control [53,54,55]. To date, there have been no reports on comprehensive management of schistosomiasis in Africa; however, comprehensive management has been attempted through the CZW project for 3 years. The measures have achieved impressive results, with schistosomiasis infection rates in some shehias now <1%, which meets the schistosomiasis elimination criteria. Hence, snail control appears to be applicable to the prevention and control of schistosomiasis in Africa and is worthy of promotion and application; however, there are also certain difficulties with the control process, such as how best to implement various control strategies in areas with different infection rates, with the aim of reducing the unnecessary use of manpower and financial resources, so that resources for schistosomiasis control can be invested in more places. These problems warrant further investigation in future studies [56].

## Figures and Tables

**Table 1:** Prevalence of *S. haematobium* infection in the Unguja and Pemba islands from 2012–2021.

Year	Unguja Island Regions							Pemba Island Regions				
	Central (%)	North A (%)	North B (%)	Urban (%)	West A (%)	West B (%)	Average (%)	Chake (%)	Micheweni (%)	Mkoani (%)	Wete (%)	Average (%)
2012	3.7	7.2	5.1	1.5	2.8	0.4	3.5	7.8	10.2	6.5	8.9	8.4
2013	8.2	14.2	5.8	1.2	3	1.9	5.7	7.1	10.3	5.1	11.3	8.5
2014	4.9	12.9	2.5	3.1	2	0.7	4.4	5.3	6.9	3.3	6.4	5.5
2015	8.6	15.6	6.2	0.7	5.9	4.3	6.9	4.2	4.9	4.8	3.7	4.4
2016	3.9	11.5	3.3	0.2	1.5	0	3.4	7.6	5.2	4	5.1	5.5
2017	1.2	5.4	1.5	0.4	1.2	0	1.6	2.1	1.5	1.2	2.1	1.7
2018	1.1	10.6	1.4	0	1.3	0	2.4	2.6	3.3	5.3	2.2	3.4
2019	0.3	2.9	0.9	0	0.2	0	0.7	1.7	0.9	1.7	2.4	1.7
2020	0.5	13.1	5.6	0	0.5	0	3.3	4.9	4	0.7	1.3	2.7
2021	4.6	7.6	3.2	0.3	1.5	0.7	3.0	2.1	0.6	1.6	2.4	1.7

**Table 2:** Polynomial regression of schistosomiasis prevalence in two islands of Zanzibar according to year.

Polynomial Regression	Island	Equation Describing the Graph	R^2^
First order polynomial	Unguja	Y = −0.334x + 676.879	0.305
	Pemba	Y = −0.768x + 1552.778	0.798
Second order polynomial	Unguja	Y = 0.0152x^2^ − 61.44x + 62,287	0.309
	Pemba	Y = 0.0894x^2^ − 361.29x + 365,052	0.867
Third order polynomial	Unguja	Y = 0.0589x^3^ − 356.28x^2^ + 718,407x − 5 × 10^8^	0.664
	Pemba	Y = 0.0018x^3^ − 10.957x^2^ + 21,913x − 1 × 10^7^	0.867

## Data Availability

All of the data supporting the findings of this study are included in the article and additional file.
